# Comparative Outcomes of Transcatheter Versus Surgical Pulmonary Valve Replacement in Adults With Congenital Heart Disease

**DOI:** 10.1002/ccd.70718

**Published:** 2026-07-01

**Authors:** Adam Bolad, Mohamed Elganainy, Fawaz Alenezi, Issam Motairek, Islam Bolad

**Affiliations:** ^1^ Indiana University Bloomington Indiana USA; ^2^ East Carolina University Greenville North Carolina USA; ^3^ Duke University School of Medicine Durham North Carolina USA; ^4^ Cleveland Clinic Cleveland Ohio USA; ^5^ Indiana University School of Medicine Indianapolis Indiana USA

## Abstract

**Background:**

Pulmonary valve replacement is frequently required in adults with congenital heart disease (CHD). Comparative real‐world outcomes between percutaneous transcatheter pulmonary valve implantation (TPVR) and surgical pulmonary valve replacement (SPVR) remain incompletely characterized. We evaluated outcomes and resource utilization for both approaches.

**Methods:**

Using the National Inpatient Sample (2016−2022), we identified adult CHD patients undergoing TPVR or SPVR. The primary outcome was in‐hospital mortality. Secondary outcomes included acute kidney injury (AKI), infection, mechanical circulatory support (MCS), and any major complication. Survey‐weighted logistic regression assessed associations with outcomes, adjusting for demographics, comorbidities, and hospital factors.

**Results:**

Among 11,285 hospitalizations (31% TPVR, 69% SPVR), overall mortality was 1.7%. Unadjusted mortality was lower for TPVR (1.0% vs. 2.0%, *p* = 0.095). After adjustment, there was no significant difference in mortality between the treatment arms (adjusted OR 2.64, 95% CI 0.92–7.61; *p* = 0.072). However, SPVR was associated with significantly higher odds of AKI (adjusted OR 5.39, *p* < 0.001), infection (adjusted OR 5.96, *p* < 0.001), and MCS requirement (adjusted OR 130.3, *p* < 0.001). Any major complication occurred in 95.5% of SPVR versus 35.4% of TPVR patients (adjusted OR 81.2, *p* < 0.001). TPVR was associated with significantly shorter LOS (2.8 vs. 9.7 days; adjusted difference −6.1 days, *p* < 0.001) and lower hospitalization charges ($209,918 vs. $336,655; *p* < 0.001).

**Conclusions:**

In adults with CHD, TPVR was associated with significantly lower complication rates, shorter hospitalization, and reduced cost compared with surgical replacement. While mortality was low for both, substantial differences in morbidity and resource utilization favor TPVR, highlighting the value of transcatheter options.

## 1 Introduction

Congenital heart disease (CHD) occurs in about 9 per 1000 live births, and about 20% of these congenital defects involve either the pulmonary valve or the right ventricular outflow tract [1]. Many of these patients with moderate to severe pulmonary stenosis or regurgitation will need surgical pulmonary valve replacement (SPVR) or transcatheter pulmonary valve replacement (TPVR) at some time of childhood or adult life [2]. SPVR is one of the most common cardiac operations in adults with CHD [3]. This reflects the large burden of late pulmonary regurgitation or stenosis after childhood repairs. About 50% of repaired tetralogy of Fallot patients required pulmonary valve replacement by the mid‐20s because of progressive pulmonary valve dysfunction [4].

Traditionally, SPVR was the only treatment available to these patients. With the progress in technology, TPVR has been used as a first‐line treatment for pulmonary valve dysfunction, especially in patients with dysfunctional right ventricle to pulmonary artery conduits or failed bioprosthetic valves [5]. In recent years, there has been an expansion of the TPVR, which allowed it to capture a large share of this patient population, which was previously surgical only. Comparative outcome data between the TPVR and SPVR is poorly characterized. We undertook this study to evaluate the real‐world outcomes in CHD patients who underwent these procedures.

## 2 Methods

### 2.1 Study Design and Population

This was a retrospective national cohort study using the National Inpatient Sample (NIS) database during a 7‐year period from 2016 to 2022. NIS is part of the Agency for Healthcare Research and Quality's Healthcare Cost and Utilization Project (HCUP). It is the largest publicly available inpatient database in the United States, capturing a random 20% stratified sample of discharges from every reporting non‐federal hospital. Each record within the database represents a single hospitalization and contains patient‐level demographics, diagnoses, and procedures.

We identified all adult hospitalizations of patients 18 years of age and older, in which TPVR or SPVR were performed utilizing the International Classification of Diseases, Tenth Revision (ICD‐10) codes beginning with “02RH.” We stratified the procedures into two mutually exclusive groups based on the fifth character of their ICD‐10 code, the character that designates the approach, to differentiate between TPVR and SPVR procedures. We excluded hospitalizations that contained codes for both TPVR and SPVR during the same hospitalization, as these cases likely represent procedural conversion or a coding error.

Patient‐level demographic variables included age (which was analyzed as a continuous and categorical variable), sex, race, insurance status, and median household income quartile for the patient's zip code. We assessed comorbidity using the Charlson comorbidity index. Comorbid conditions identified as covariates to adjust for included pulmonary hypertension, right ventricle failure, atrial fibrillation, chronic kidney disease, diabetes mellitus, and liver disease. Resource utilization was also evaluated, including length of stay, hospital charges, and discharge disposition.

### 2.2 Study Endpoints

The primary endpoint was in‐hospital mortality; secondary endpoints included acute kidney injury (AKI), acute myocardial infarction, acute heart failure or cardiogenic shock, infection/sepsis, vascular complications, arrhythmia requiring intervention, mechanical circulatory support (MCS), and repeat interventions. A composite major complication endpoint was defined as the occurrence of any of the above complications or mortality during hospitalization.

### 2.3 Statistical Analysis

All analyses accounted for the sampling design of NIS by applying the provided discharge weights and stratification variable included in the dataset, in addition to the HOSP_NIS variable as the primary sampling unit, as this variable links in‐patient records to their respective hospital. Utilizing this approach was pertinent to our study design as it yields the nationally representative estimates and corrects standard errors using Taylor‐series linearization.

Baseline characteristics between TPVR and SPVR patients were compared for the descriptive analysis using survey‐weighted linear regression for the continuous variables and design‐based *F*‐tests for the categorical variables. To assess the independent association between procedure type and outcomes, we created survey‐weighted logistic regression models for binary outcomes and survey‐weighted linear regression models for continuous outcomes. The primary independent variable was procedure type. All models were adjusted for previously identified comorbidities, sex, race, age, year of admission, Charlson comorbidity index, and hospital characteristics. Results were reported as adjusted odds ratios (OR) with 95% confidence intervals. To verify the outcomes of our study, we performed a subgroup sensitivity analysis for the primary outcome, stratified by age category, pulmonary hypertension status, RV dysfunction status, and the presence of chronic kidney disease. A *p* value of < 0.05 was considered statistically significant. All analyses were performed using STATA version 19 statistical software.

## 3 Results

**Figure 1:**
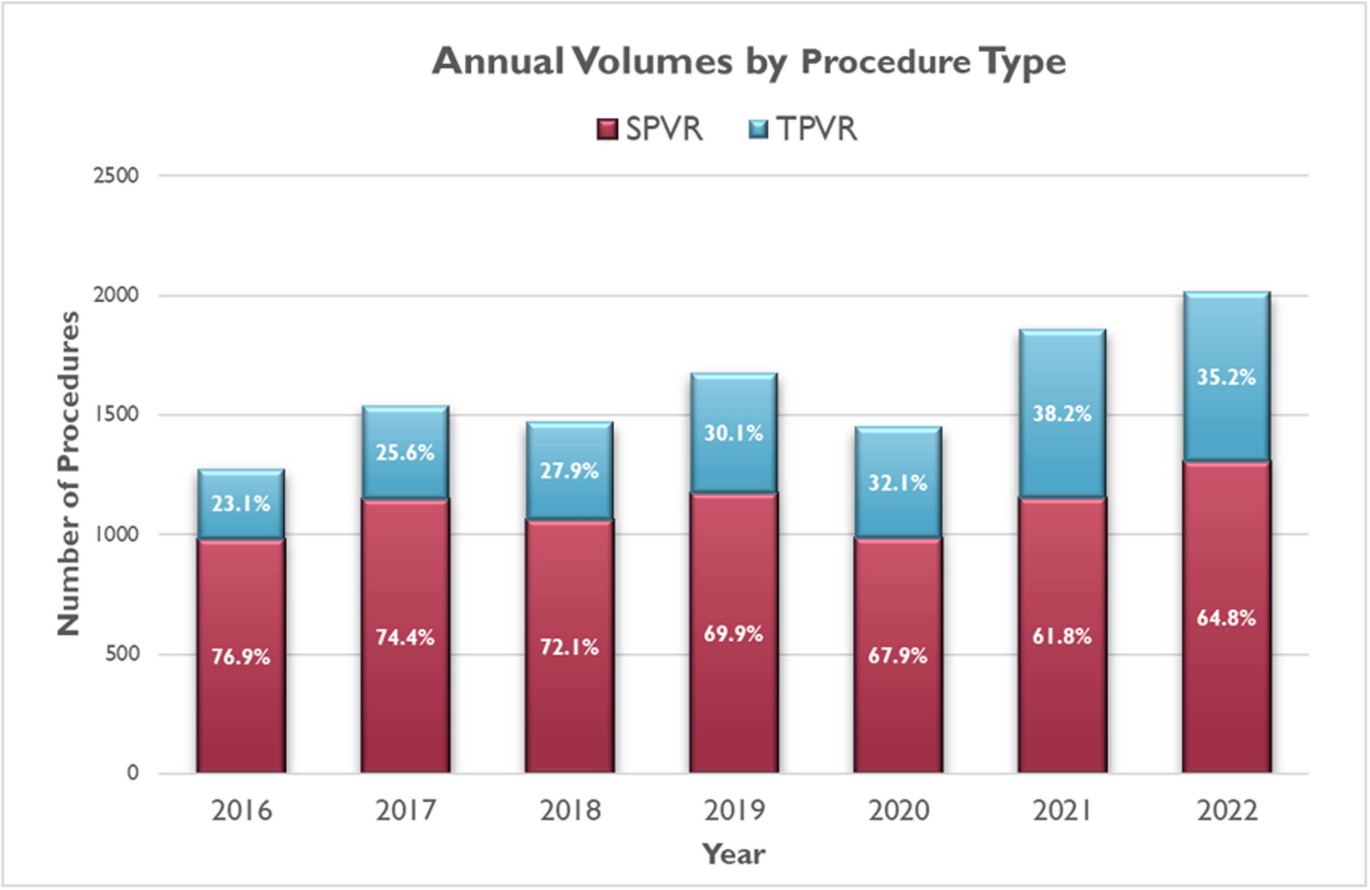
Annual unweighted volume and proportion of TPVR versus SPVR procedures in the United States, 2016−2022. [Color figure can be viewed at wileyonlinelibrary.com]

During the 7‐year study period from 2016 through 2022, there were 11,285 pulmonary valve procedures in adults with CHD. Overall, TPVR was performed in 31% of patients, and SPVR was performed in 69% of these patients. The volume of these procedures increased over the study period, as shown in Figure 1. TPVR procedures increased from 23.1% in 2016 to 38.2% procedures in 2021. Total procedures nearly doubled, increasing from 1275 procedures in 2016 to 2015 procedures in 2022.

Table 1 shows the baseline characteristics of patients undergoing TPVR and SPVR. There were 3493 TPVR and 7792 SPVR procedures. The mean age of admission was similar between groups: 38.0 versus 38.9 in TPVR and SPVR, respectively. Further, the age categories between groups did not differ significantly. TPVR had more female patients (45.6% vs. 40.9%, *p* = 0.039) and a lower comorbidity burden by Charlson index score (0.98 vs. 1.13; *p* = 0.027). The usage of insurance was different between groups (*p* = 0.001), as TPVR showed a higher proportion of Medicaid and Medicare (23.4% vs. 20.9%) and a lower proportion of private insurance compared to SPVR (53.6% vs. 63.2%). Diabetes was a more frequent comorbidity among TPVR patients (11.8% vs. 9.0%; *p* = 0.027), while the other comorbidities analyzed did not differ significantly. We found that there was a significant difference between hospital region (*p* < 0.001) and admission year (*p* = 0.012).

**Table 1:** Baseline characteristics of adults undergoing pulmonary valve replacement.

Characteristic	TPVR (*n* = 3493)^a^	SPVR (*n* = 7792)^a^	*p* value
*Demographics*			
Age, years, mean ± SE	38.0 ± 0.6	38.9 ± 0.4	—b
Age category, %			0.131
18–30	38.4	35	
31–40	21.4	22.6	
40–49	16.9	15.1	
≥ 50	23.4	27.3	
Female sex, %	45.6	40.9	0.039
*Race/ethnicity, %*			0.075
White	68.1	73.3	
Black	12	8.6	
Hispanic	12.9	10.3	
Asian	3	3	
Other	0.5	0.6	
Missing	3.6	4.2	
*Insurance status*			
Insurance type, %			< 0.001
Medicare	20.8	13.9	
Medicaid	23.4	20.9	
Private	53.6	63.2	
Self‐pay	2.3	2.1	
*Median household income quartile, %*			0.057
Q1 (lowest)	26.5	22.2	
Q2	25.4	24.7	
Q3	24.8	25	
Q4 (highest)	23.3	28.1	
*Comorbidities*			
Charlson comorbidity index, mean ± SE	0.98 ± 0.05	1.13 ± 0.04	0.027
Pulmonary hypertension, %	11.9	10.7	0.381
Right ventricular dysfunction, %	1.7	1.9	0.821
Atrial fibrillation, %	18.5	20.7	0.24
Chronic kidney disease, %	6.9	6.8	0.946
Diabetes mellitus, %	11.8	9	0.045
Liver disease, %	2.3	3.3	0.204
*Hospital characteristics*			
Hospital region, %			< 0.001
Northeast	14.9	22.1	
Midwest	26.5	28.3	
South	29.7	29.4	
West	28.9	20.2	
*Hospital bed size, %*			0.69
Small	5.4	6.5	
Medium	12.5	11.9	
Large	82.1	81.5	
Teaching hospital, %	98.7	98	0.21
Year of admission, %			0.012
2016	8.5	12.6	
2017	11.3	14.7	
2018	11.8	13.6	
2019	14.5	15	
2020	13.3	12.6	
2021	20.3	14.8	
2022	20.3	16.7	

*Note:* All proportions and *p* values are survey‐weighted. *p* values from design‐based *F* tests for categorical variables.

^a^ Weighted national estimates using NIS discharge weights. Unweighted sample: TPVR, *n* = 630; SPVR, *n* = 1627. Total adult analytic cohort: *n* = 2257 (weighted *n* = 11,285);

^b^ Age was compared using survey‐weighted linear regression.

The in‐hospital mortality and secondary outcome measures are shown in Table 2. In‐hospital mortality was not clinically significant between TPVR and SPVR (adjusted OR 2.64, 95% Cl 0.92–7.61). AKI (5.9% vs. 17.9%; OR 5.39), MCS/ECMO (9.7 vs. 91.6%, OR 130.33), and infection/sepsis (1.2% vs. 5.5%; OR 5.96) were significantly more frequent in SPVR. The composite of any major complication was substantially higher in SPVR (35.4% vs. 95.5%; OR 81.24). Resource utilization favors TPVR as the length of stay was substantially shorter (3.0 vs. 9.6 days) and total charges were significantly less ($209,918 vs. $336,665).

**Table 2:** In‐hospital outcomes of TPVR versus SPVR in adults with congenital heart disease.

Outcome	TPVR (%)	SPVR (%)	Unadj. OR (95% CI)	Adj. OR^a^ (95% CI)
*Primary Outcome*				
In‐hospital mortality	1	2	2.00 (0.87–4.60)	2.64 (0.92–7.61)
*Secondary Outcomes—Complications*				
Acute kidney injury	5.9	17.5	—b	5.39 (3.47–8.36)^e^
Acute MI	0.3	0.8	—	3.02 (0.72–12.11)
Acute HF/cardiogenic shock	30.7	36.8	—	1.15 (0.88–1.51)
Infection/sepsis	1.2	5.5	—	5.96 (2.41–14.70)^e^
MCS/ECMO	9.7	91.6	—	130.33 (89.24–190.34)^e^
Vascular complications	0^c^	0.1	—	7.46 (1.08–51.36)^d^
Repeat intervention	0	0.9	—	—f
Any major complication (composite)	35.4	95.5	—	81.24 (52.93–124.69)^e^
*Resource utilization*				
	Mean ± SE	Mean ± SE		Adj. Coeff.^g^ (95% CI)
Length of stay, days	3.0 ± 0.2	9.6 ± 0.3	—	6.08 (5.35–6.81)^e^
Total charges, $	209,918 ± 5894	336,655 ± 9514	—	101,256 (77,693–124,818)^e^

Abbreviations: CI = confidence interval, ECMO = extracorporeal membrane oxygenation, HF = heart failure, OR = odds ratio, MCS = mechanical circulatory support, MI = myocardial infarction, SPVR = surgical pulmonary valve replacement, TPVR = transcatheter pulmonary valve replacement.

^a^ Adjusted for age, sex, race/ethnicity, income quartile, Charlson comorbidity index, pulmonary hypertension, right ventricular dysfunction, atrial fibrillation, chronic kidney disease, diabetes, liver disease, hospital teaching status, region, bed size, insurance type, and year of admission;

^b^ Unadjusted ORs not shown for secondary outcomes; adjusted ORs reported directly;

^c^ Fewer than 11 weighted events; proportion suppressed per HCUP guidelines;

^d^ *p* < 0.05;

^e^ *p* < 0.001;

^f^ Model did not converge due to complete separation (0% events in TPVR group);

^g^ Adjusted coefficient from survey‐weighted linear regression; represents the adjusted difference for SPVR versus TPVR (reference).

Table 3 shows the multivariate survey‐weighted logistic regression for in‐hospital mortality, and Table 4 shows the sensitivity analysis. In the fully adjusted mortality model, SPVR had an OR of 2.64 (95% Cl 0.92−7.61; *p* = 0.072). Increasing age was associated with higher odds of in‐hospital mortality (OR: 1.03 per year; *p* = 0.027). Pulmonary hypertension (OR 3.13; *p* = 0.015) and liver disease (OR 25.007; *p* = 0.001) have a strong association with In‐hospital mortality. Certain hospital factors had an affiliation with increased risk of mortality: living in the South compared to the Northeast (OR 4.02, Cl 1.14−14.18; *p* = 0.030). There was a decreased risk of in‐hospital mortality in medium bed size hospitals compared to small bed size hospitals (OR 0.13, Cl 0.02–0.91; *p* = 0.040). We performed a sensitivity analysis that excluded repeat procedures as seen in Table 4. SPVR association was similar (OR 2.71, 95% Cl 0.94−7.83; *p* = 0.065), proving the consistency of our findings.

**Table 3:** Multivariable survey‐weighted logistic regression for in‐hospital mortality.

Variable	OR	95% CI	*p* value
SPVR (vs. TPVR)	2.64	0.92–7.61	0.072
Age (per year)	1.03	1.00–1.06	0.027
Female sex	1.32	0.61–2.89	0.481
*Race/ethnicity (vs. White)*			
Black	0.55	0.13–2.24	0.401
Hispanic	0.63	0.19–2.08	0.447
*Income quartile (vs. Q1)*			
Q2	0.95	0.30–2.99	0.929
Q3	2.54	0.93–6.95	0.07
Q4 (highest)	0.89	0.26–3.03	0.853
Charlson comorbidity index	1.13	0.86–1.48	0.396
Pulmonary hypertension	3.13	1.25–7.84	0.015
Right ventricular dysfunction	0.82	0.05–13.84	0.889
Atrial fibrillation	1.96	0.84–4.66	0.127
Chronic kidney disease	1.2	0.35–4.19	0.77
Diabetes mellitus	0.92	0.28–3.02	0.896
Liver disease	25.77	9.81–67.73	< 0.001
Teaching hospital (vs. non‐teaching)	0.25	0.05–1.21	0.086
*Hospital region (vs. Northeast)*			
Midwest	1.23	0.31–4.87	0.766
South	4.02	1.14–14.18	0.03
West	1.51	0.34–6.61	0.584
*Hospital bed size (vs. small)*			
Medium	0.13	0.02–0.91	0.04
Large	0.7	0.15–3.18	0.644

*Note:* Model also adjusted for insurance type and year of admission (not shown). Survey design accounted for NIS complex sampling.

Abbreviations: CI = confidence interval, OR = odds ratio.

**Table 4:** Sensitivity analysis for in‐hospital mortality.

Analysis	OR	95% CI	*p* value
Primary analysis (fully adjusted)^a^	2.64	0.92–7.61	0.072
Excluding repeat procedures^b^	2.71	0.94–7.83	0.065

Abbreviations: CI = confidence interval, OR = odds ratio for SPVR relative to TPVR (reference).

^a^ Adjusted for age, sex, race/ethnicity, income quartile, Charlson comorbidity index, pulmonary hypertension, atrial fibrillation, CKD, diabetes, liver disease, teaching status, region, bed size, insurance, and year.

^b^ Excludes patients with repeat interventions during the same hospitalization (*n* = 21 unweighted).

Table 5 shows the discharge disposition of unweighted discharge counts and percentages by procedure type. Routine discharge to home was more common after TPVR than SPVR (96.1% vs. 80.0%). Use of home health care was substantially more frequent in SPVR than TPVR (14.2% vs. 1.4%), and transferring facilities (3.5% vs. 0.8%) were more common in SPVR than TPVR.

**Table 5:** Discharge disposition by procedure type (unweighted).

	TPVR		SPVR	
Disposition	*n*	%	*n*	%
Routine (home)	983	96.1	2158	80
Short‐term hospital transfer	10	1	18	0.7
Other transfers^a^	8	0.8	94	3.5
Home health care	14	1.4	382	14.2
Against medical advice	1	0.1	0	0
Died in the hospital	7	0.7	47	1.7
Total	1023	100	2699	100

*Note:* Counts are unweighted; includes all ages.

^a^ Includes skilled nursing facility, intermediate care facility, and other facility transfers.

## 4 Discussion

Our results showed that TPVR was associated with lower overall mortality compared to SPVR, although the incidence did not reach statistical significance. However, SPVR was associated with a higher incidence of AKI, infection, and MCS requirement. Also, SPVR was associated with a significantly higher incidence of any major complication. TPVR was associated with significantly shorter LOS and lower hospitalization charges.

TPVR avoids sternotomy and cardiopulmonary bypass in adults with CHD, who often have multiple prior operations and significant comorbidities [6]. Our study showed that TPVR patients have a trend toward lower mortality, although it did not reach statistical significance. Our results are consistent with findings from meta‐analyses and large database studies of TPVR consistently showing lower in‐hospital or composite early mortality and major morbidity post TPVR compared to SPVR in patients with similar baseline CHD complexity. In a meta‐analysis of 11 studies which included about 4300 patients, the OR for in‐hospital mortality was 0.18 favoring TPVR compared to SPVR [7]. TPVR is performed via venous catheters and avoids sternotomy and cardiopulmonary bypass which reduces surgical trauma and blood loss and systemic inflammation. On the other hand, SPVR, specially redo sternotomy in scarred mediastinum, involves cardiopulmonary bypass and extensive dissection, which increase the risk of low output syndromes, arrhythmias and multiorgan failure that could contribute to the in‐hospital death. In addition, TPVR is associated with shorter procedure time and less need for transfusion in addition to shorter ICU and hospital LOS. This lower burden of peri‐procedural complications translates into lower in‐hospital mortality [6]. In contemporary practice, TPVR allows for treatment of dysfunctional conduits and bioprosthetic valves without the need for another open‐heart surgery, thus avoiding the risk of multiple sternotomies and cardiopulmonary bypass. Single‐center studies sometimes find similar early mortality between TPVR and SPVR after baseline differences are accounted for, suggesting that some of the observed advantages may reflect case selection. However, large meta‐analyses studies show a small but measurable reduction in in‐hospital mortality and better outcomes overall compared to SPVR [8], a finding similar to what we found in our national database study. Central illustration 1

**Central illustration 1:**
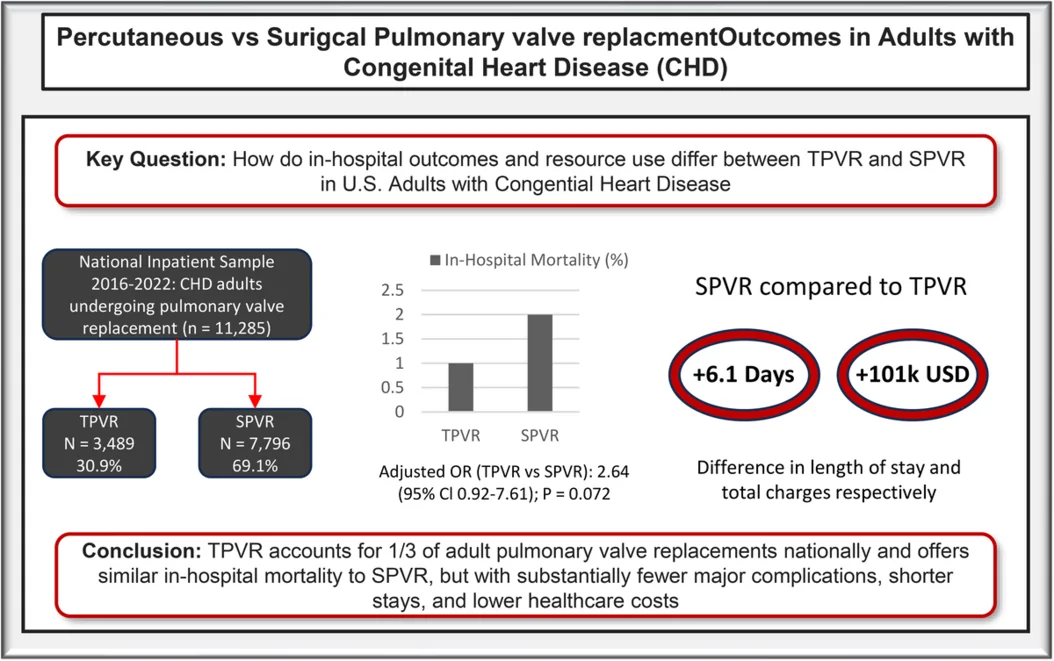
Among 11,285 US adults with congenital heart disease undergoing pulmonary valve replacement (NIS 2016–2022), transcatheter replacement (TPVR) was associated with similar in‐hospital mortality but significantly fewer major complications, 6.1 fewer hospital days, and approximately $101,000 lower total charges compared to surgical replacement. [Color figure can be viewed at wileyonlinelibrary.com]

In our study, patients with CHD who underwent SPVR sustained a significantly higher incidence of AKI compared to those who underwent TPVR. This is likely because surgery requires cardiopulmonary bypass and aortic cross‐clamping, which leads to hemodynamic stress and a direct negative effect on the kidneys, whereas TPVR avoids the cardiopulmonary process [9]. Cardiopulmonary bypass with non‐pulsatile flow and hemodilution causes renal hypoperfusion, inflammation, and microembolization, all of which are associated with AKI. In addition, longer cardiopulmonary bypass and cross‐clamp times, which are needed for more complex procedures in patients with CHD are associated with postoperative AKI [10]. Also, open heart surgery which involves greater intraoperative blood loss and low cardiac output predispose to the renal ischemia‐perfusion injury leading to AKI [11]. Extrapolating from large aortic valve cohorts where AKI was significantly less after transcatheter than surgical replacement supports the plausibility that avoiding cardiopulmonary bypass and open heart surgery reduces the risk of AKI in CHD patients undergoing TPVR [12]. On the other hand, TPVR is associated with use of iodinated contrast, which generally has a negative effect on the kidneys. However, this is a relatively brief and is due to tubular and vasoconstrictive insult, and usually occurs in patients with pre‐existing chronic kidney disease, use of higher contrast dose, and with repeated exposure to iodinated contrast [13, 14]. Even though TPVR uses contrast and can trigger postprocedural AKI, current data suggest that when the contrast volume use is limited and the patient is hydrated, the risk is low compared with the multifactorial AKI risk of the cardio‐pulmonary bypass used during cardiac surgery [14]. For CHD patients undergoing TPVR with carefully managed contrast volume, renal insults are low compared to SPVR with cardiopulmonary bypass, and that explains why AKI rates are generally lower after TPVR despite contrast use [9].

We found that SPVR is associated with increased incidence of in‐hospital infection compared to TPVR in the post‐procedure period. This is likely because SPVR requires sternotomy and cardiopulmonary bypass in addition to mechanical ventilation, in addition to a longer intensive care unit and hospital stay [7]. All these factors increase exposure to sternal infection, in addition to exposure to central line infection and ventilator‐associated pneumonia. TPVR requires shorter procedure time and length of hospital stay, leading to less time in high‐risk environments which in general lowers the postoperative infection risk [15]. Our study shows that TPVR is advantageous for early recovery and non‐valve infection during the hospital admission for the index procedure. This aligns with the findings of published meta‐analyses [16]. This may or may not translate to reduced risk of long‐term infection, including valve infection [17].

The use of MCS after SPVR was significantly higher than after TPVR in our CHD patient population. The use of cardiopulmonary bypass after SPVR causes systemic inflammatory response and temporary myocardial dysfunction. This can be deleterious in situations where the heart struggles to maintain adequate function following the bypass, most commonly occurring in long cross‐clamp times and pre‐existing ventricular dysfunction in CHD patients [18]. In these instances, MCS use, such as ECMO, may be needed to support the circulation during the recovery period. Many CHD patients underwent previous surgeries, making redo sternotomies difficult. This increases the risk of complications, including injury to cardiac structures during dissection of the extensive adhesions from previous surgeries, requiring MCS support to stabilize the patient [7].

In‐hospital LOS was significantly shorter for TPVR compared to SPVR. TPVR is performed percutaneously through a small incision in the groin to access the femoral vein or a small incision in the neck to access the jugular vein, whereas SPVR requires a sternotomy. By avoiding the large surgical incision with the subsequent need for wound healing, TPVR patients experience significantly reduced risk of site‐specific complications, allowing for early hospital discharge [7]. The LOS in our patient population is 2.8 days for TPVR compared to 9.7 days for SPVR, and it is similar to the published data [8]. In addition, the difference in LOS in patients undergoing TPVR compared to SPVR of 6.1 days is larger than what is reported in meta‐analyses, in which the difference was found to be about 4–5 days [7]. This increased LOS benefit in our study is perhaps due to improved technology and increased experience in performing TPVR in patients with CHD.

The hospitalization charges in TPVR were significantly lower in our study compared to SPVR. Each procedure involves costs in different areas. TPVR has higher devices and procedures, and supply costs. The transcatheter pulmonary valves and delivery systems are expensive and are generally more expensive than the surgical bio‐prosthesis. In SPVR, the cost is driven by the operating room and the cardiac team, ICU admission, and the longer LOS. In our study, there was overall cost saving benefit in using TPVR. Review of the literature shows that this is not the case everywhere, and TPVR can incur similar or maybe higher cost compared to SPVR [19]. The device and TPVR procedure sometimes offset savings from fewer hospital days. It is possible that with increased utilization of TPVR, the percutaneous pulmonary valve and supplies pricing has come down, in addition to savings from the improved utilization and efficiency in performing this percutaneous procedure, due to increased experience.

Our findings in this national study comparing TPVR to SPVR in CHD patients is generally similar to what has been reported in meta‐analyses. TPVR has opened new horizons for this high‐risk CHD patient population, most of whom have already undergone multiple surgeries. We see that as the technology continues to progress, and the TPVR procedure is adopted and widely performed by more centers and individuals, TPVR might be performed as the first‐line therapy for CHD patients requiring pulmonary intervention, similar to the current adoption of transcatheter (TAVR) rather than surgical (SAVR) aortic valve replacement for managing aortic valve disease in most patients needing aortic valve intervention.

## 5 Limitations

This is a retrospective study based on routine patient clinical care and treatment with TPVR and SPVR. This study design inherently introduces selection bias. The decision to proceed with either TPVR or SPVR is driven by unmeasured anatomical factors. TPVR is generally reserved for patients with favorable anatomy, whereas SPVR is the standard for native or complex right ventricular outflow tracts that are too large for transcatheter devices. TPVR patients are often high surgical risk patients, and many of them have multiple prior sternotomies and are sicker, whereas SPVR patients often include patients undergoing elective repair for other lesions. There is always a risk of inaccurately coding CHD patients, including the risk of lumping together CHD lesions. In addition, there is a risk of failing to capture the complexity of prior repairs, as the history of prior cardiac surgery does not specify how many surgeries the patient had or what type of surgeries they had [20]. This makes it difficult for the two different treatment options to have comparable baseline cardiac anatomy.

Despite these limitations, our study has multiple strengths. Our study has a strong advantage in sample size as it includes thousands of CHD patients across many centers, which allowed us to study these relatively uncommon TPVR and SPVR procedures with adequate power for endpoints such as in‐hospital mortality, AKI, infection, and use of MCS [21]. This large sample size allows us to reflect better on real‐world practices of using TPVR and SPVR, better than single‐center series [22]. With such a large sample size, we were able to perform subgroup analyses such as age and payer to see however outcome differs across the CHD subpopulations [23]. Our database allows a robust means of recording in‐hospital mortality reliably following TPVR and SPVR within the index hospitalization. In addition, our secondary endpoints, such as the LOS and hospital charges, are core elements of our national database, allowing detailed comparison of resource utilization and economic impact of TPVR compared to SPVR in CHD patients [19]. Because our database is nationally representative, our findings can assist resource allocation and guideline implementation [21]. Our findings can serve as a platform for hypothesis generation and trial design for future prospective studies [22].

## 6 Conclusions

Contemporary practice in the United States indicates that although both TPVR and SPVR in CHD patients have low mortality, TPVR shows a trend of lower in‐hospital mortality compared to SPVR in this patient population. Compared to SPVR, our study showed that TPVR was associated with significantly less incidence of AKI, in‐hospital infection, and MCS requirement. In addition, TPVR was associated with significantly shorter LOS and lower hospitalization charges. Despite the difference in resource utilization and morbidity, further research is needed in the form of clinical trials with rigorous patient selection criteria to understand differences in clinical outcomes, resource utilization and quality of life assessments between those two treatment strategies in this complex CHD patient population.

## Funding

The authors have nothing to report.

## Ethics Statement

This research was adherent to the practices required by the Agency for Healthcare Research and Quality. Institutional review board approval and patient consent were not required as the analysis was based on de‐identified publicly available databases.

## Conflicts of Interest

The authors declare no conflicts of interest.

## Data Availability

The National Inpatient Sample (NIS) data used in this study is publicly available from the Healthcare Cost and Utilization Project at http://www.hcup-us.ahrq.gov/, through the Agency for Healthcare Research and Quality.
